# Immunosensing Based on Optical Fiber Technology: Recent Advances

**DOI:** 10.3390/bios11090305

**Published:** 2021-08-30

**Authors:** Maria Simone Soares, Miguel Vidal, Nuno F. Santos, Florinda M. Costa, Carlos Marques, Sónia O. Pereira, Cátia Leitão

**Affiliations:** Physics Department & I3N, University of Aveiro, 3810-193 Aveiro, Portugal; msimone.fsoares@ua.pt (M.S.S.); miguelvidal@ua.pt (M.V.); nfsantos@ua.pt (N.F.S.); flor@ua.pt (F.M.C.); sonia.pereira@ua.pt (S.O.P.); catia.leitao@ua.pt (C.L.)

**Keywords:** biosensor, optical fiber, biofunctionalization, surface plasmon resonance (SPR), antibody

## Abstract

The evolution of optical fiber technology has revolutionized a variety of fields, from optical transmission to environmental monitoring and biomedicine, given their unique properties and versatility. For biosensing purposes, the light guided in the fiber core is exposed to the surrounding media where the analytes of interest are detected by different techniques, according to the optical fiber configuration and biofunctionalization strategy employed. These configurations differ in manufacturing complexity, cost and overall performance. The biofunctionalization strategies can be carried out directly on bare fibers or on coated fibers. The former relies on interactions between the evanescent wave (EW) of the fiber and the analyte of interest, whereas the latter can comprise plasmonic methods such as surface plasmon resonance (SPR) and localized SPR (LSPR), both originating from the interaction between light and metal surface electrons. This review presents the basics of optical fiber immunosensors for a broad audience as well as the more recent research trends on the topic. Several optical fiber configurations used for biosensing applications are highlighted, namely uncladded, U-shape, D-shape, tapered, end-face reflected, fiber gratings and special optical fibers, alongside practical application examples. Furthermore, EW, SPR, LSPR and biofunctionalization strategies, as well as the most recent advances and applications of immunosensors, are also covered. Finally, the main challenges and an outlook over the future direction of the field is presented.

## 1. Introduction

Since the 1970s, when the extraordinary revolution in optical fiber (OF) technology took place, extensive research has been dedicated to this area. As a result, optical fibers broadened their use from optical transmission waveguides in telecommunications to sensing devices for different applications, namely for monitoring temperature, mechanical strain, refractive index (RI), pressure and concentration of analytes [1,2]. This means that optical fibers can be used in a variety of fields ranging from environmental monitoring [3] to biomedical diagnosis [4] and food safety [5], since these waveguides present high flexibility and compactness, as well as the ability for remote measurement [6] and immunity to electromagnetic interference [7].

In an optical fiber, the RI of the core is slightly higher than that of the surrounding cladding, allowing light to propagate and remain confined within the core through total internal reflection (TIR) [8]. In this case, the light ray strikes the boundary interface with an incidence angle larger than the critical angle, according to Snell’s law, and the light is totally internally reflected and propagated through the fiber. As a result, losses to the surroundings are minimal when the light rays are guided through the fiber core. Nonetheless, a small portion of light penetrates the reflecting medium, being designated by evanescent wave (EW) [9]. This wave has an intensity that decreases exponentially to zero in the cladding before reaching the external medium. These principles are beneficial for telecommunications yet hinder local RI sensing functions due to the lack of interaction between light and the surroundings. To use the EW in biosensing, it is crucial to enhance its interaction with the external medium. For this reason, several methods have been employed to enable this interaction, including geometric modifications to the fiber and the inscription of diffraction gratings in the fiber core [7].

Optical sensing mechanisms include absorption, fluorescence and surface plasmon resonance (SPR), among others [7]. In the first case, no coatings or metal layers are used for the detection of analytes. Consequently, detection is accomplished through the direct interaction of the EW with the molecules of interest. In the second case, usually a coating with photoluminescence properties is used, in which fluorescence arises when the EW interacts with the coating. In the SPR mechanism, photons from a light source interact with surface electrons of a metal layer and lead them to oscillate collectively. The excitation of these surface plasmons (SPs) can result in the propagation of surface waves at the metal-dielectric interface (surface plasmon waves—SPWs). When a strongly localized oscillation of surface electrons takes place in nanoparticles (NPs), it is designated as localized SPR (LSPR).

The first plasmonic sensors were fabricated based on the Kretschmann configuration using prism structures. However, the bulkiness of these prism-based biosensors poses a disadvantage and reduces its applicability for in situ biosensing [8]. The first fiber optic sensor based on SPR was proposed by Jorgenson and Yee in 1993 [10]. From that time on, SPR fiber optic biosensors attracted considerable attention and achieved promising results [11]. Therefore, these biosensors have been used in the most diverse areas of research as they can overcome issues associated with conventional SPR biosensors and potentially add the following characteristics: tolerance to harsh environments, remote and distributed sensing [8].

Traditional detection methods, such as enzyme-linked immunosorbent assay (ELISA) and polymerase chain reaction (PCR), despite their high sensitivity and broad use, involve time-consuming and expensive procedures that need to be performed in adequate facilities by skilled operators [12,13,14]. As such, research and development of new diagnostic devices that are cost-effective, rapid and portable in a way that can be used in the point of care (POC) are crucial, in which optical biosensors based on optical fibers are included.

In general, biosensors are devices that comprise three main components: the bioreceptor, the transducer and the signal readout processor. The bioreceptor is the element responsible to provide a specific biorecognition event for a target analyte, whereas the transducer transforms this biochemical reaction in a measurable signal that is processed by the signal processor. They can be classified according to the biorecognition element, which can be based on catalytic (enzymes, cells or tissues) [15] or affinity interactions, namely antibodies (ABs) or nucleic acids [16]. The current review will focus on immunosensors, which are biosensors that employ antibodies as bioreceptors.

Biosensors, and in this specific case, immunosensors, can be also classified according to the transducing mechanism, which can include electrochemical, piezoelectric, optical and thermometric [17], among others. Particularly, optical biosensors have been studied extensively since they offer direct, real-time and on-site detection compared to conventional analytical techniques [18]. In all cases, immunosensors can be further divided in labelled and label-free operation. In the former, a label is incorporated, which is used to identify the formation of the immunocomplex (the antigen−antibody complex) through the measurement of the label signal. On the other hand, in label-free immunosensors, the formation of the analyte−antibody complexes is measured by monitoring the consequent physical changes of the transducer, without adding any labels [16].

**Figure 1:**
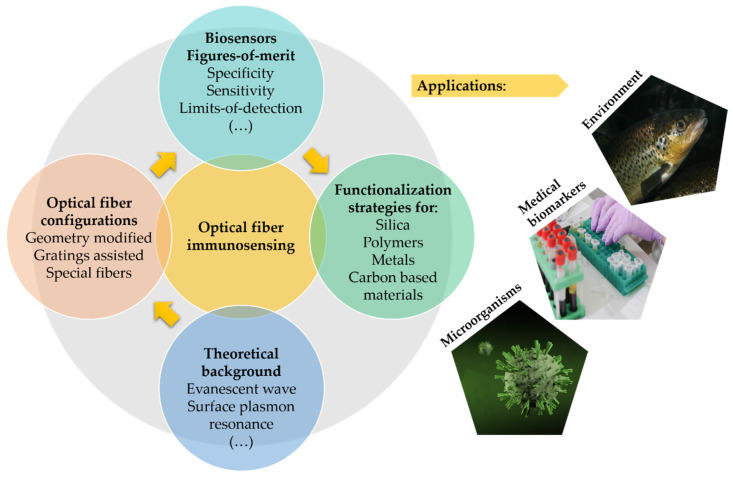
Schematic illustration of the main contents on this review.

The purpose of this review is to outline the different geometries and configurations used in optical fiber biosensors over the years and provide information about biofunctionalization strategies and their working principles, including EW and SPR/LSPR with reference to plasmonic materials. Lastly, a section on the state-of-the-art immunosensors is also covered. The contents of this paper are schematically summarized in Figure 1.

## 2. Theoretical Background on Optical Fiber Biosensing Working Principles

### 2.1. Evanescent Wave

**Figure 2:**
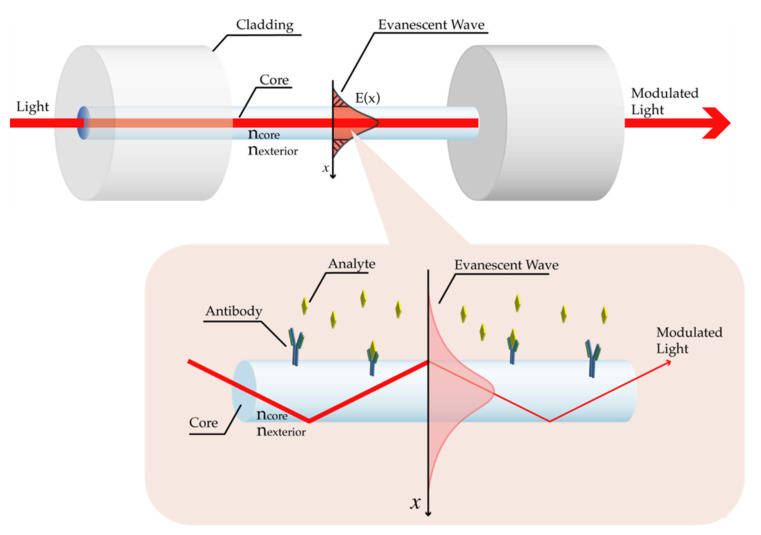
Schematic illustration of the TIR phenomenon in an uncladded optical fiber, with the resulting EW representing the EW reaching of the AB-analyte interaction and consequent modulation of optical signal by the surrounding RI change.

The light propagating in a fiber can be divided into the guided field in the core (core modes) and the evanescent wave, created at the TIR location in the core-cladding interface, that decays exponentially to zero (cladding modes), as schematically illustrated in Figure 2 [7,19]. Moreover, the short distance that the EW travels in the low RI medium (cladding) is called the penetration depth and corresponds to the distance where the evanescent field decreases to 1/*e* of its initial value at the interface [19]. According to [20], the penetration depth can have values between λ/3 and λ/2. The detection of analytes of interest can be accomplished in the area reached by the penetration depth, through the interaction of the EW with those molecules.

When the EW interacts directly with the external medium in the presence of analytes, light can be absorbed by the atoms or molecules that constitute the analyte sample. Consequently, absorption phenomena can be used to quantify samples and concentrations of analytes since the absorption spectra differ for different analytes. Therefore, optical fiber biosensors based on absorption phenomena are highly dependent on the external medium RI [21].

As previously mentioned, optical fiber biosensors can also rely on labels, such as fluorophores. Fluorescence can occur in the presence of polyaromatic hydrocarbons or heterocycles, also designated by fluorophores or fluorescent dyes. When the analyte sample labelled by these chemical components is stroked by the EW, light is absorbed and the chemical components are excited, leading to the emission and detection of photons at a longer wavelength [21]. Properties such as light intensity, decay time, polarization or phase of the emitted radiation can be used to evaluate the concentration of the analyte [9]. This sensing mechanism not only presents fast response, but also high sensitivity and specificity as well as good reproducibility [22].

To allow a better interaction of the EW with the surrounding medium and, consequently, an improvement of analyte detection, several optical fiber structures were fabricated and studied as described in Section 2.3.

### 2.2. Surface Plasmon Resonance and Localized Surface Plasmon Resonance

The SPR principle was introduced to the field of biosensing by Liedberg et al. [23] in 1983 and is currently the mechanism behind most optical fiber biosensors in the field of biomedicine. This optical detection technique has been extensively used as it allows label-free monitoring of biomolecular interactions in real time with fast response, high sensitivity and very low limit of detection (LOD) [24,25].

The SPR arises when light traveling through the dielectric medium hits the metal surface under the resonance condition of the SPs. The resonance or phase-matching condition represents the situation when the propagation constants of the incident light and the SPs are equal and, therefore, their momentum match and resonance take place. For this reason, SPR is very sensitive to RI changes [24], providing the ability to monitor biochemical reactions occurring at the metal−dielectric interface [26].

Several designs have been proposed to promote SPR excitation, each resorting to different coupling devices such as prisms [27,28], waveguides [29,30,31,32] and gratings [33,34]. Among the existing methods, the Kretschmann−Raether configuration remains the most popular in commercial systems nowadays. In this approach, light is injected through a high RI glass prism towards its metal-coated base, usually with gold (Au) or silver (Ag), with an incidence angle greater than the critical angle so that TIR occurs. This generates an EW that propagates along the interface separating the metal film and the surrounding dielectric medium [26,35]. If the propagation constant of the EW matches that of the SP, thereby fulfilling the resonance condition, the wave can transfer energy to the SP [36], leading to a decrease of the reflection at a given angle [26]. Hence, monitoring the reflectance of the SPW can be used to screen changes of the surrounding environment. Nevertheless, these prism-based sensors are expensive and bulky, which hinders in situ biosensing, particularly in locations of difficult access [7,8]. For these reasons, vast research has been devoted to optical fiber technology as it provides a highly-integrated and miniaturized structure compared to their bulky counterparts, with greater flexibility in terms of design, materials and performance, along with the capacity for in vivo measurement [8,37].

In optical fiber SPR-based sensors, the light guided in the fiber core must be exposed to the surrounding medium so that it can interact with metal coatings or particles placed on the fiber in order to elicit SPR. While the first reported optical fiber plasmonic sensors resorted to removal of the cladding, numerous other techniques and structures have emerged since then, as described in the following sections. In these structures, spectral and intensity interrogation techniques are generally used, by measuring wavelength shifts and optical power changes in the transmission or reflection spectra [26,36].

In addition, LSPR-based sensors have also been the focus of extensive research, having demonstrated promising performance results [38,39]. In this optical detection technique, the incident light waves are confined within conductive NPs, whose dimensions are inferior to the wavelength of the light. Similarly to SPR, the excitation of localized SPs (LSPs) arises from the interaction of the incident light and the electrons in the conduction band of the metals, inducing strong localized oscillations of surface electrons [24,36]. Despite having the same excitation mechanism, contrary to SPR, which involves lossy propagation along the intersection between metal and dielectric nanofilm, LSPs are non-propagating and oscillate locally in the nanostructure as a result of particle size restriction [7,8,37]. For LSPR, detection relies on monitoring the change of the absorbed wavelength of the light propagating through NPs dispersed in liquids or deposited on solid substrates. Once RI changes occur in the area surrounding the NPs, the resonant wavelength shifts in accordance [36]. Furthermore, the resonant frequency is highly dependent on the dielectric environment, distance between particles and intrinsic properties of the metal NPs, namely composition, size, shape, geometry and aspect ratio [17,40]. Therefore, tuning these factors can optimize the detection performance towards a specific target [27]. Some benefits of LSPR include enhanced light intensity at the resonant wavelength and high spatial resolution, and the use of NPs offers a larger surface contact area than continuous films, allowing more analytes to bind to the metals [41,42]. However, attaining reproducibility of sensing fabrication is difficult as a consequence of particle size, shape and aspect ratio constraints [42].

Different plasmonic materials have been deposited onto optical fiber biosensors for both SPR and LSPR, with Au and Ag being the most commonly used [8] for nanofilms and NPs [24,36]. Between the two, Au demonstrates a larger shift of the resonance peak, whereas Ag evidences a narrower peak width and fewer optical losses. However, Ag oxidizes easily once exposed to aqueous environment, unlike Au which is chemically inert and, therefore, much more stable [42]. The deposition of a thin bimetallic layer onto the Ag surface, prior to application in biological systems, can inhibit oxidation but leads to a decrease in sensor performance [43]. The performance of plasmonic biosensors is highly influenced by the shape of the deposited metal particles and the characteristics of the coating, namely morphology, thickness (usually 30–70 nm) and rugosity [26]. Loyez et al. [44] conducted a study to evaluate the influence of Au thickness (35, 40, 45 and 50 nm) on the sensors’ sensitivity, which increased with thickness from 808.4 nm·RIU^−1^ (35 nm) to 1650.2 nm·RIU^−1^ (50 nm), however, with an increase in variability. For thicknesses below 35 nm and beyond 50 nm, deep SPR responses could not be verified. Nevertheless, reproducibility of the deposition process was considered weak, seeing as, for 50 nm, only 60 % of optrodes presented a correct SPR resonance. Moreover, the SPR wavelength position was also affected by the Au thickness.

Over the years, graphene and its derivates, namely graphene oxide (GO) and reduced GO (rGO), started being included in plasmonic optical fiber sensors. Graphene is a mechanically strong and chemically inert two-dimensional (2D) carbon allotrope, with a zero-band gap [25,43] and a hexagonal lattice structure that prevents the passage of oxygen molecules and thus inhibits oxidation [42,45]. Several studies have proven that the addition of a graphene layer to a metal surface improves sensitivity [24,43]. As an example, in 2017, Jiang et al. developed an LSPR biosensor using a U-shape POF covered with an Ag film, a layer of polyvinyl alcohol, graphene and Ag NPs. Their study revealed that graphene improved sensitivity, reaching 700.3 nm·RIU^−1^, while also delaying the oxidation process of the Ag NPs, maintaining the sensor’s stability [46]. Plasmonic biosensors can also be based on GO as it provides several benefits including high biocompatibility, colloidal stability and easy chemical modification of the surface oxide. GO comprises a large amount of oxygen-containing functional groups that aid biomolecules’ immobilization through covalent bonding. Additionally, the chemical modification of the surface can be used to regulate these groups in order to optimize the energy band gap and thus enhance the SPR coupling effect [25]. Qi Wang and Bo-Tao Wang [47] proposed a SPR-based optical fiber biosensor for detection of human immunoglobulin G (IgG). In this study, using an Ag film and a GO/Ag coating, sensitivities to RI of 2875 nm·RIU^−1^ and 3311 nm·RIU^−1^ were respectively achieved. This increase was attributed to the intensity enhancement of the confined electric field surrounding the sensing layer as a result of adding the GO film. Other alternative materials are emerging in this field, including molybdenum disulfide (MoS_2_) [48], zinc oxide (ZnO) [49], titanium dioxide (TiO_2_) [50] and titanium nitride (TiN) [51].

The materials’ science research delivered refined coating techniques of optical fibers with different metals and semiconductors. The possibility of changing the materials’ properties such as thickness, shape and structure as well as addition of nanostructures can tune the spectrum resonance wavelength, increase the intensity of SPs, improve optical fibers’ RI sensitivity and ultimately improve the detection accuracy and sensitivity to analytes [52].

### 2.3. Optical Fiber Configurations for Biosensing

Optical fibers can be divided into silica optical fibers (SOFs) and polymeric optical fibers (POFs) [53]. Nowadays, these types of fibers are widely applied in sensors. POFs, usually made of a poly(methylmethacrylate) (PMMA) core and a fluorinated polymer cladding [54], have also been used for the development of biosensors owing to recent advances in POF technology. Besides, POFs can withstand smaller bend radii in contrast to SOFs [2]. In addition, regarding the number of modes that can be transmitted, optical fibers can have different designations. Light propagates inside the optical fiber as an electromagnetic wave, and this wave can propagate in a single mode—single-mode fibers (SMFs), or in several modes—multimode fibers (MMFs). The number of modes that can propagate in the core depends not only on the diameter of the fiber, but also on the wavelength of the radiation [41]. Usually, for the development of biosensors, SMF SOFs or MMF POFs are applied. SMF SOFs generally have a cladding diameter of 125 μm, a core diameter of 9 μm and a numerical aperture value of 0.13 [55]. On the other hand, MMF POFs have cladding and core diameters of 1000 μm and 980 μm, respectively, with a numerical aperture value of 0.5 [56].

In a standard optical fiber, the incident light is confined within the core region, as a result of TIR, if the incidence angle of the light is higher than the critical angle [9]. This provides very little losses to the surroundings, which is fundamental for fiber-optic telecommunications, but prevents interaction of the light with the fiber coatings and analytes. For this reason, several modifications of the fiber, with distinct production processes and costs, have been developed to enable biosensing functions [7]. These include geometry-modified sensors, grating-assisted sensors and special fibers. Some of the most common operating principles of these structures are based on EW and SPR/LSPR, in which a metal coating is applied. Several strategies have been adopted to improve RI sensitivity of optical fibers by disrupting the light propagation in the core, resulting in different configurations and sensing mechanisms, as will be described in the next sub-sections.

#### 2.3.1. Geometry-Modified

**Figure 3:**
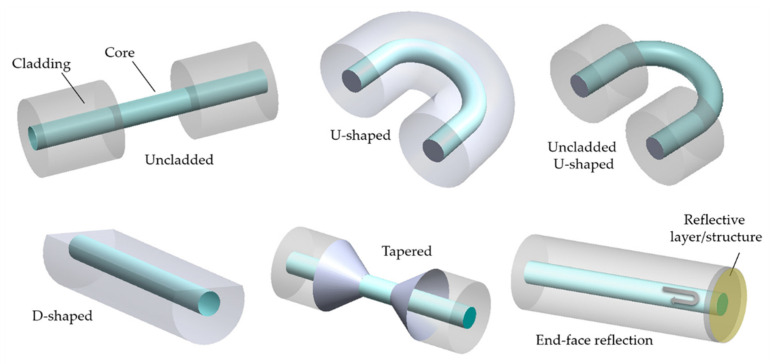
Schematization of geometry-modified OF configurations for biosensing.

There is a considerable number of geometry-modified configurations which include: uncladded, U-shaped, D-shaped, tapered and end-face reflected, as schematized in Figure 3.

For the production of unclad optical fibers, the fiber cladding can be mechanically removed by the aid of a sharp blade [32], especially in the case of POFs [8], or through chemical etching [57,58], mainly applied in SOFs. Another approach consists in bending the fiber to obtain a U-shaped structure, which allows coupling from the core mode to the cladding modes, enhancing the penetration depth of the evanescent field and consequently the fiber sensitivity [59,60]. In a study, Khijwania and Gupta [61] concluded that sensitivity increases with the decrease of bending radius until a certain critical value, referred to as optimum bending radius, beyond which the fiber becomes lossy and sensitivity decreases. This value depends on the numerical aperture and the core radius of the fiber.

D-shaped fibers are one of the strategies used as biosensors and are usually manufactured by side-polishing or micromachining. In order to obtain a D-shaped sensing structure, it is necessary to remove part of the cladding only on one side of the fiber, unlike unclad or etched fibers. Therefore, this structure presents a flat side-wall that allows easy deposition of a material of interest that can hence interact with the light guided in the core [62].

Tapered sensing structures are also used in biosensors and can be accomplished by two methods: both core and cladding of the fiber are tapered, or the cladding is removed and only the core is tapered [19]. In both cases, the fiber is stretched along axial direction while heating it over a heat source, decreasing the diameter of the fiber (usually several micrometers). After incident light enters the tapered region, the incident angle decreases gradually and approaches the critical angle of total reflection, where part of the incident light eventually leaks into the cladding. Thus, the evanescent field is exposed to the surroundings, and both the evanescent field magnitude and penetration depth increase [63]. In other words, when light propagates through the tapered region, it gets distributed into lower- and higher-order modes that are supported by the core and cladding, respectively. The power of the higher-order modes is easily leaked into the surrounding medium in the form of an EW [64].

All the aforementioned structures are based on light transmission, whereas the end-face reflected configuration relies on reflection at the end of the fiber. Sometimes, to improve the sensor’s sensitivity, a portion of the fiber tip is uncladded before the application of the reflective layer. Thus, once incident light reaches this layer, it is reflected backwards, doubling the optical path and enhancing the response of the sensing region as a result [44,65].

Comparing the different presented geometries, it should be noted that the selection of the geometry to apply will depend on the final application. For instance, fibers in the U-shape, despite presenting easy manufacture, result in bulky sensors which can limit their use in applications with low volume analyte solutions. However, due to their repeatability and easy production, they can be an attractive solution for other suitable applications. For in situ measurements, such as inside the human body, end-face reflection geometries represent the best choice, since low volumes of solutions are needed and the reflection configuration represents a great advantage in this context. Uncladded, D-shaped and tapered geometries result in small sized biosensors, usually interrogated in transmission, that can be used in miniaturized photonic chips. A challenge for these geometries can be their structural repeatability, meaning it could be difficult to define a manufacturing process that guarantees sensors to always have the same and the desired performance.

#### 2.3.2. Grating-Based

An optical fiber grating is a kind of fiber device with a permanent period change of RI on the fiber core and was demonstrated by Ken Hill et al. [66] in 1978. After forty years of development, the fabrication and commercialization of fiber gratings has progressed swiftly. These gratings fall into two general classifications based on the grating period: short period gratings (period less than 1 μm) and long period gratings (LPGs) [11,67,68]. The information provided by the grating is encoded in wavelength and, as this is an absolute parameter, the optical signal can be processed in a way that the information remains immune to energy fluctuations along the optical path [68].

**Figure 4:**
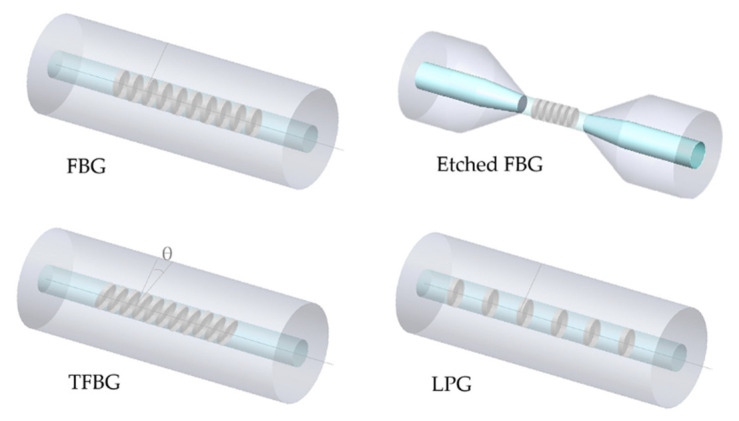
Schematization of grating-based OF configurations.

Furthermore, grating-modified configurations can be distinguished into uniform fiber Bragg gratings (FBGs), etched FBGs, tilted FBGs (TFBGs) and LPGs as will be briefly presented below and displayed in Figure 4.

FBGs are short period gratings and, initially, were fabricated through a visible laser whose light propagated along the fiber core. However, in 1989, Gerald Meltz and his coworkers demonstrated a much more flexible technique of transverse holographic inscription, in which the core was exposed to ultraviolet (UV) laser light through the cladding [69]. This technique uses the interference pattern of UV laser to create the periodic structure of the Bragg grating in the fiber. After decades of active research and development, FBGs have been used for sensing in a large scale. In addition, the progress of laser technology allowed the possibility of inscribing high quality gratings using femtosecond lasers [70,71].

As follows, FBGs consist of a set of narrowband reflectors, having equally spaced grating patterns, inscribed on the optical fiber. The grating period defines the wavelength of the light that is reflected, and the periodic perturbations in the sensors based on FBGs act essentially as wavelength filters. Although many wavelengths are reflected at each perturbation, the majority disappear by destructive interference with other reflected signals. Only a specific wavelength undergoes constructive interference. As a result, a narrowband spectrum is reflected, csentered on a central wavelength known as the Bragg wavelength (*λ_B_*). This condition is expressed as [19]:

(1) $$\lambda_{B}=2n_{eff}\Lambda$$

where *n_eff_* is the effective RI of the FBG and Λ is the grating period. In summary, FBGs allow the coupling of light from the forward-propagating mode to a backward (counter propagating mode) at a specific wavelength, the Bragg wavelength [72].

As mentioned above, the operating principle of an FBG is based on the dependence on effective RI and grating period. In a standard FBG, the effective RI is not influenced by the external RI, because coupling only happens between core modes [11,68]. However, if the cladding diameter is reduced along the grating region, the transmitted light in the core is significantly affected by the external RI. This means that the lower the diameter in the region containing the grating, the higher the sensitivity. Nevertheless, this reduction entails higher fragility and more difficulties in fiber handling [73,74]. Consequently, changes in the Bragg wavelength combined with a modulation of the reflection amplitude are expected to occur. In this case, the FBG becomes sensitive to the surrounding RI changes occurring in the measurand [73]. Different methods can be used to reduce the cladding, such as etching, grinding and fine-drawing cone [11].

TFBGs are short period (~500 nm) gratings with a periodic pattern uniformly tilted by an angle θ (<45°) with respect to the fiber axis, leading to a more complex coupling compared to uniform FBGs [26,74]. In addition to the self-backward coupling of the core mode at the Bragg wavelength, TFBGs also redirect some light to the cladding, which allows coupling to circularly and non-circularly symmetric co-propagating or counter propagating cladding modes. The direction of the coupled light depends on the tilt angle of the TFBG [26,74]. For this reason, the transmitted spectrum shows multiple narrow attenuation bands that are related to the coupling with different cladding modes. In this case, the Bragg condition is slightly modified because it takes into account the resonance wavelengths of the various cladding modes ($\lambda_{res\left(m\right)}^{TFBG}$) given by [74]:

(2) $$\lambda_{res\left(m\right)}^{TFBG}=\left(n_{eff}^{core}-n_{eff}^{clad\left(m\right)}\right)\frac{\Lambda }{\cos\left(\theta \right)}$$

where $n_{eff}^{core}$ and $n_{eff}^{clad\left(m\right)}$ are the effective RIs of the core and the *m*^th^ cladding mode, respectively. Conforming to the phase-matching condition, when the effective RI of the corresponding cladding modes is identical to the surrounding RI, maximum sensitivity is achieved [11]. Besides this, as a consequence of phase matching, the spectral position of a resonance depends on the effective RI of the corresponding cladding mode, which in turn depends on the optical properties of the medium close to the cladding surface [26]. Thus, when the surrounding RI increases, the resonance wavelengths of the corresponding cladding modes shift to longer wavelengths [74]. A benefit of using TFBGs relies on their sensitivity to temperature and strain, that can be independently monitored, providing control over undesired fluctuations [26].

On the other hand, in LPGs, the grating period typically ranges from 100 µm to 700 µm and coupling occurs between the fundamental core mode and co-propagating cladding modes. For this reason, multiple attenuation bands at discrete resonance wavelengths are present in the transmission spectrum, with each band related to coupling to a specific cladding mode. These resonance wavelengths, $\lambda_{res\left(m\right)}^{LPG}$, satisfy the phase-matching condition expressed by [7,74]:

(3) $$\lambda_{res\left(m\right)}^{LPG}=\left(n_{eff}^{core}-n_{eff}^{clad\left(m\right)}\right)\Lambda$$

Considering that the cladding modes propagate near the interface between cladding and surrounding medium, they are sensitive to changes in the RI of the latter [26]. As such, once the surrounding RI increases, the resonance wavelengths shift to shorter values [74]. Moreover, LPGs are sensitive to bending, axial strain and temperature, which can cause unwanted spectral fluctuations [26,75].

In regard to the selection of the best grating approach for a final application, once again, it depends on the context and interrogation systems available. From all gratings, TFBGs and LPGs usually present higher RI sensitivity, resulting in sensors with low LODs and high sensitivities. However, at this moment, they require the availability of optical spectrum analyzers (OSAs), which are high-cost and bulky equipment. Nonetheless, this should not be a reason for a divestment in these types of sensors, as several researchers are simultaneously working on new technologies for interrogating fiber optic sensors, which can overcome this limitation soon. As for to FBGs, they present a lower RI sensitivity; however, due to the higher simplicity in interrogation, as they present only one narrow peak at the Bragg wavelength, they can be the best option for analytes and contexts where high sensitivity is not required.

#### 2.3.3. Special Fibers

In order to overcome the limitations of conventional fibers, special optical fibers have been proposed and investigated. Micro-structured optical fibers (MOFs), structures that have air holes or micro-capillaries inside the fiber, have been used owing to their higher degree of integration, longer interaction distance between light and analyte, which occurs along the entire length of the fiber, and improved robustness. Furthermore, in MOFs, the fabrication of the sensing region happens inside of the fiber instead of on the exterior, which allows effective interaction between the analyte of interest and the evanescent field of the core mode [37,76].

In recent years, a variety of MOF structures have been proposed, such as hexagonal, semicircular channel and exposed core, among others. Moreover, when air holes in the MOFs present periodic formation (periodic microstructure), usually in the region of the coating and along the entire length of the fiber, they are known as photonic crystal fibers (PCFs) [77,78]. These can be divided into solid-core PCFs, which operate based on a TIR mechanism, and hollow-core PCFs, in which the guiding mechanism depends on the material that is placed at the core site [8]. PCFs can further be classified according to the external structure in, for example, D-shape [51] and H-shape [79]. In most MOFs, the main objectives are to facilitate analyte infiltration and provide larger interaction area [37].

**Figure 5:**
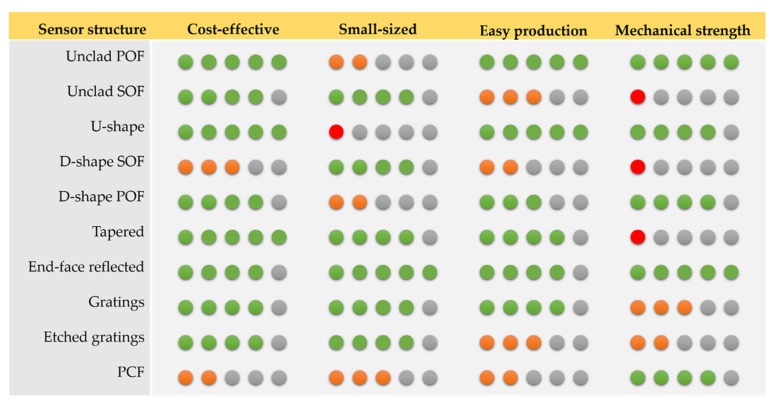
Infographic of the main characteristics of several optical fiber structures (green: great–good; orange: medium–fair; red: poor).

Figure 5 presents a comparison of some of the main characteristics of the aforementioned optical fiber structures (geometry-modified, grating-modified and special fibers), namely in terms of cost, size, ease of fabrication and mechanical strength.

### 2.4. Figures-of-Merit of Biosensors

The performance of a biosensor is validated by assessing their figures of merit, which include sensitivity, specificity, limit of blank (LOB), limit of detection (LOD), limit of quantitation (LOQ), linear or working concentration range, accuracy, repeatability and reproducibility (Table 1). Sensitivity (*S*) corresponds to the ratio between the change in the output signal (for instance, in optical biosensors, wavelength or intensity) in response to variations of the concentration of the measured analyte. This Figure-of-merit is obtained from the slope of the linear calibration curve of the biosensor. Specificity denotes the ability to discern the response of the analyte of interest from every other interferent. LOB represents the highest apparent analyte concentration expected to be found when replicates of blank samples are tested (with no analyte present in the sample) [80]. On the other hand, LOD is the lowest analyte concentration, distinguished from the noise or LOB, detectable by the sensor with acceptable degree of certainty, but not necessarily quantified [81]. LOQ is the lowest analyte concentration that can be quantified with an acceptable degree of certainty. In the literature, different methods have been applied to calculate the LOD [82], which is a critical problem concerning the performance comparison of biosensors; thus, it is highly suggested that the method for LOD calculation should be always present. The first and more straightforward way is by visual definition from the regression line [82]. Others use the calibration curve, if available, for LOD calculation according to Equation (4), when no background noise is present and a blank response is inexistent [83]. In this case, the LOD is defined by:

(4) $$LOD=\frac{k\sigma }{S}$$

where *k* is a numerical factor that depends on the level of confidence (usually *k* = 3 is used of LOD calculation whereas *k* = 10 for LOQ); σ is the standard deviation (SD) of the y-intercept or residual SD of the linear regression; and *S* is the sensitivity given by the slope of the linear regression.

Another method, due to the presence of background noise in the response or because the blank response (samples without the presence of analyte) provides an analytical signal, takes into account the blank (Equation (5)) or LOB (Equation (6)) for LOD determination [80,82]. The former uses the mean apparent concentration of the blank and the SD of blank sample (*σ_blank_*) and, in the second approach, first the LOB is calculated (Equation (7)) followed by LOD determination (Equation (8)) [80,84]:

(5) $$LOD=mean_{blank}+k\sigma_{blank}$$

(6) $$LOB=mean_{blank}+1.645\sigma_{blank}$$

(7) $$LOD=LOB+1.645\sigma_{low\;concentration\;sample}$$

In certain cases, the LOD of optical measurements can be calculated as the ratio between the spectrometer wavelength resolution (Δλ) and the sensor sensitivity [47,85]:

(8) $$LOD=\frac{\Delta \lambda }{m}$$

Furthermore, accuracy refers to the proximity between the sensor output value and the actual measured value. Finally, repeatability and reproducibility designate the sensor’s capability to obtain a similar response over several repetitions using the same analyte, with the former considered when performing under the same conditions (operators, equipment, laboratories and intervals of time, among others) and the latter under different conditions [36,81].

## 3. Biofunctionalization Strategies for Optical Fiber Immunosensors

**Figure 6:**
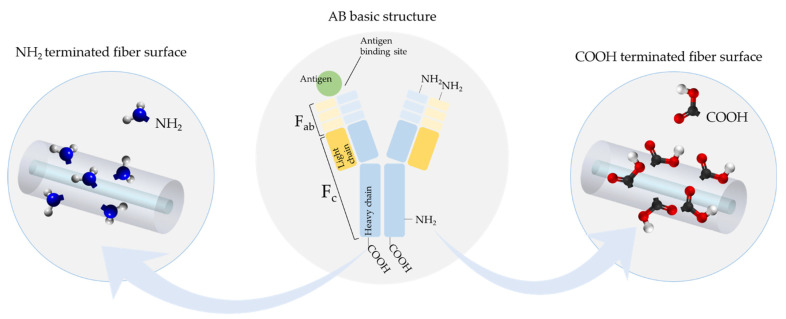
Schematic representation of the antibody structure (**center**) and the representation of amine (**left**) and carboxylic (**right**) functional groups onto the fiber surface used for antibody immobilization.

Immunosensors are one of the main classes of biosensors owing to the highly specific and selective immunoreaction that takes place once an antibody recognizes and binds to a specific antigen [16]. The development of an optical fiber immunosensor requires the immobilization of antibodies on the fiber surface, a step that has a crucial impact on sensor performance. Therefore, knowing the structure of an antibody is of major importance. An antibody is a Y-shaped glycoprotein that belongs to the immunoglobulin (Ig) superfamily and is composed of the following regions, as schematically represented in Figure 6: a fragment crystallization (Fc) region and two fragment antigen binding (Fabs) regions in which the specific antigen binding site is located [86].

**Figure 7:**
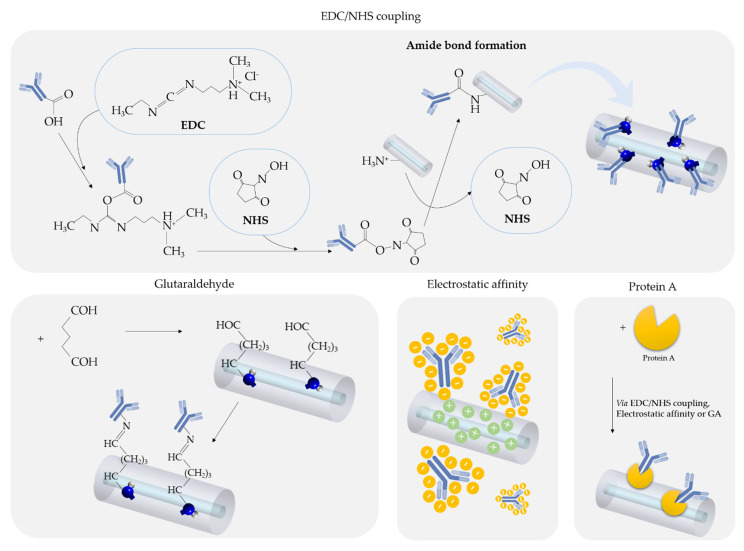
Schematic representation of antibody immobilization strategies: using EDC/NHS coupling or GA via electrostatic interaction or using Protein A.

The main aspects to be considered regarding the immobilization of antibodies include the orientation and density, along with the preservation of the biological activity of the antibody. The density, i.e., number of antibodies per unit surface area, regulates the efficiency and prevents non-specific interactions with undesirable biomolecules, and either lower or higher antibody densities could result in poor sensing performance. Few studies dedicated their efforts to the impact of the surface density of immobilized antibodies in the sensors’ performance; for deeper understanding, refer to [87,88,89]. On the other hand, the orientation determines the availability of the antigen binding sites to bind the antigens, specifically [86]. In this review, we focused on the antibody orientation, taking into consideration the different functionalization approaches that have been applied. For instance, the Fab region possesses the amine terminal, and this functional group should be avoided during antibody immobilization so that the antibody is not unavailable for antigen binding. Therefore, antibodies are usually covalently linked by their carboxylic groups in the Fc region in order to assure that the Fab region is available for the antigen recognition event [90]. Furthermore, the chemical nature of the fiber as well as the presence and nature of surface coating have to be taken into account during the selection of the proper biofunctionalization strategy. Therefore, two main consecutive steps can be considered for the preparation of the immunosensing layer onto an optical fiber: (i) surface modification with an intermediary linker, and (ii) the linkage of the antibody. As concerns the former, the chemical nature of the surface fiber (bare or coated) will define the intermediary linker that will be used, as presented and discussed in the following sub-sections. The result of this step is a surface terminated with functional groups such as amines or carboxylic groups, as represented in Figure 6, but other functional groups can also be promoted. Regarding the second step, different immobilization strategies, as represented in Figure 7, namely covalent immobilization or via electrostatic interaction, can be applied, in which their choice also depends on the functional groups provided by the intermediary linker in the first step.

The two most popular bioconjugated reagents to yield a covalent linkage are the zero-length crosslinker strategy using 1-ethyl-3-(3-dimethylaminopropyl)carbodiimide hydrochloride (EDC), or glutaraldehyde (GA), which is a homobifunctional crosslinker. EDC activates carboxylic groups promoting the formation of amide bonds when in the presence of amine groups. The efficiency of this reaction can be enhanced by using *N*-hydroxysuccinimide (NHS) [91]. Usually, in order to guarantee an adequate orientation of the antibody, the amine (-NH_2_) groups presented in the fiber surface are bonded to the carboxylic (-COOH) group belonging to the antibody terminal in the Fc region [84,92], whereas using GA, aldehyde groups from GA react with amine groups either from the fiber surface or from antibodies to promote their covalent linkage through imine bonds [91,93].

The antibody can be physically adsorbed on the fiber surface via electrostatic interactions. IgG antibodies with the isoelectric point at slightly acidic pHs have a global negative charge at physiological conditions (pH = 7.4); hence, one can take advantage to immobilize it onto surfaces with a positive charge. This strategy is the easy and simple one, but it leads to a random orientation of the antibody on the surface, so the antigen-binding sites might be unavailable for the formation of the antibody-antigen complex.

Alternatively, Protein A can be used as an intermediate protein to guarantee a proper orientation of the antibody as it recognizes and bonds to the Fc region of IgG antibodies, hence the Fab region stays available. Protein A can be immobilized on the fiber surface either via covalent linkage [54] or via electrostatic interactions [94]. Although the correct orientation of the antibody is an advantage in this strategy, it requires more functionalization steps and can produce sensors with lower sensitivity as the antibodies are far from the fiber surface [94].

Lastly, the biofunctionalization procedure ends with a passivation step. The most common passivating agents are bovine serum albumin (BSA) [13,31,47,54,95,96,97,98] and poly(ethylene glycol) (PEG) [99]. These reagents are commonly implemented to reduce the non-specific interactions between the analyte and surface, and also between interferents present in more complex matrixes.

### 3.1. Bare Silica Optical Fiber

**Figure 8:**
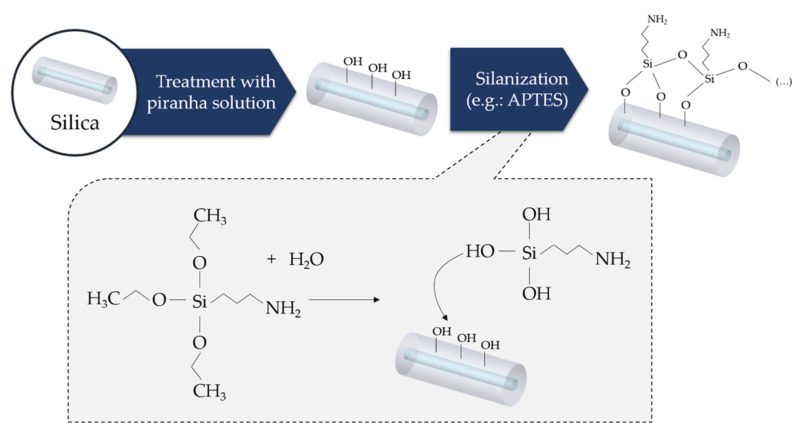
Schematic representation of an SOF surface modification using a silane as an intermediary linker to produce an amine-terminated surface.

Vast research has been performed regarding the immobilization of antibodies on SOFs, with oriented or site-directed immobilizations garnering increasing attention [100]. In this case, a common approach consists in, firstly, treating the glass surface using a piranha solution aiming to introduce reactive hydroxyl groups on the fiber surface. This step is followed by a silanization process that can be achieved using different silanes based reagents, for instance, (3-aminopropyl)trimethoxysilane (APTMS) [94], -(3-aminopropyl)triethoxysilane (APTES) [93,101,102], (3-mercaptopropyl)trimethoxysilane (MTS) [103], [3-(triethoxysilyl)propyl]succinicanhydride (TESPSA) [104], (3-glycidoxypropyl)trimethoxysilan (GLYMO) [105] and 3-aminopropyl(diethoxy)methylsilane (ADMS) [106,107]. The most used silanes are APTES and APTMS, which yield surfaces with free amine groups (-NH_2_) that can be later used to covalently link the antibodies, either using EDC/NHS or GA bioconjugation reagents. Figure 8 depicts the silanization procedure on a silica optical fiber.

Considering the physical adsorption strategy via electrostatic immobilization, as aforementioned, the antibodies possess a negative surface charge at physiological pH conditions, thus the fiber must have a positive surface charge. SOFs can be treated with NaOH in order to produce a negative surface charge and, subsequently, a polycation such as poly(allylamine) [108] or poly-l-lysine [109] is used to produce a positively charged surface. Alternatively, the immobilization of antibodies via electrostatic interaction can be promoted by directly immersing the silanized optical fiber on the antibody solution [94].

In 2018, Loyez et al. [94] compared three functionalization strategies to immobilize anti-cytokeratin 17 (CK17) antibodies on glass TFBGs. First, APTMS was used as intermediary agent, and then the authors explored (i) the antibody immobilization using GA for covalent binding (ii) via electrostatic interaction and (iii) using Protein A. According to their results, while all can be effective for biosensing, the electrostatic adsorption proved to be the fastest and most effective. In detail, while covalent bonding evidenced larger modal shifts for higher CK17 concentrations, electrostatic adsorption showed a better LOD (14 pM) and constant shifts between each concentration. The affinity method employing Protein A, however, evidenced slightly lower modal shifts and higher measurement errors, which was attributed not only to the greater distance between the target and the fiber surface but also to the complexity of the functionalization process [94].

Moreover, in a 2020 study by Janczuk-Richter [104], several surface modification methods were assessed, namely, physisorption on a clean surface, physisorption on APTES-modified surface, covalent bonding to APTES, covalent bonding to TESPSA and covalent bonding to EDC-activated TESPSA. The physisorption methods were the least efficient, with the first evidencing slightly better results compared to the APTES-modified approach. While all the techniques using covalent bonds lead to great surface coverage, the last two showed the best effects, resulting in the highest surface distribution. Between these two, the TESPSA method was preferred due to its greater simplicity. In this approach, proteins were immobilized through amide bonds between the succinic anhydride group in TESPSA and the amine groups in the protein.

### 3.2. Plastic Optical Fiber

**Figure 9:**
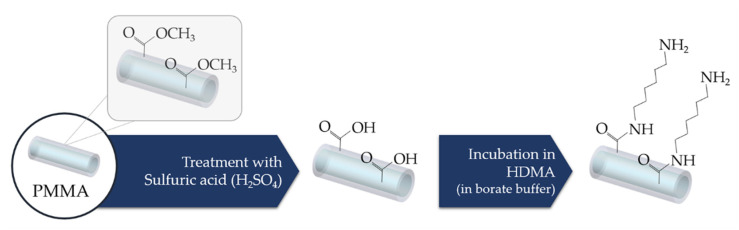
Schematic representation of a POF surface modification to produce an amine-terminated surface.

The functionalization process in an unclad POF whose surface is of pure PMMA can be achieved by reacting PMMA with hexamethylenediamine (HMDA), yielding an aminated surface [54,95]. First, the sensors are treated with sulfuric acid (H_2_SO_4_) to reduce methyl ester (-COOCH_3_) groups through acid hydrolysis, producing carboxylic groups on the POF surface. Afterwards, the fiber is incubated in HMDA in order to form amine groups on the hydrolyzed surface [38,54,99], becoming more hydrophilic. The amine-terminated fibers can be then functionalized with the antibody by direct covalent linkage to the surface using GA [99] or using the intermediary Protein A [54], as depicted in Figure 9.

### 3.3. Metal-Coated Fibers

**Figure 10:**
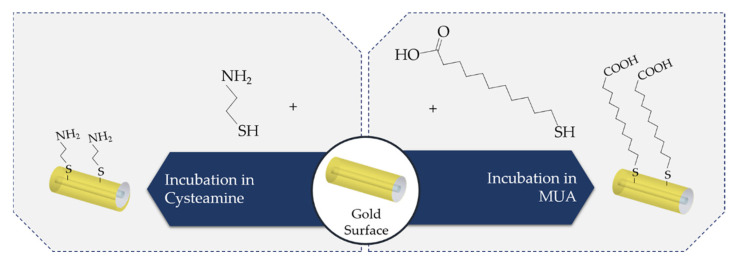
Schematic representation of a gold-coated fiber modified with cysteamine or MUA to produce amine-terminated or carboxylic-terminated surfaces, respectively.

In many studies, Au is the chosen metal to trigger SPR as well as Au NPs to create LSPR. Cysteamine is one of the intermediary linkers used to immobilize the antibody on the Au surface. Therefore, one approach consists in, firstly, immersing the sensors in a cysteamine solution to bind the thiol (-SH) functional groups of the cysteamine molecule to the Au surface through a strong affinity interaction. Secondly, the antibodies are covalently immobilized through the carboxylic acid (-COOH) functional groups to the cysteamine -NH_2_ functional groups in the fiber surface, using EDC/NHS bioconjugate reagents [13,31,84,92], as schematized in Figure 10.

Another approach utilizes Protein A covalently bonded to Au NPs modified with 11-mercaptoundecanoic acid (MUA). This linker has a thiol group, which strongly binds to gold, and, in the other terminal, a carboxylic group, which in turn can be activated by the EDC/NHS chemistry and covalently linked to amine groups from Protein A [96]; see Figure 10. In this case, this strategy presented better sensitivity and lower limits of detection when compared with the same antibody directly linked to MUA-modified Au NPs. [96].

In 2020, Chang et al. [110] reported a competitive assay for the development of a fiber-based biosensor. The competitive assay occurs between free analyte in solution, in this case, methamphetamine (MA), and MA conjugated with BSA immobilized on the surface. To do so, the Au NP-coated fiber was immersed in an ethanol solution of 6-mercapto-1-hexanol (MCH) and MUA to create a mixed self-assembly monolayer. The following step was the activation of the -COOH groups from MUA through EDC and NHS chemistry and immobilization of MA-BSA on the surface of the sensor. In this case, to deactivate the unreacted sites, ethanolamine was used [110]. In other studies, dopamine is used as the cross-linking agent instead of cysteamine and Protein A. In this case, the antibody is immobilized on the surface of poly(dopamine)-coated fiber [30].

### 3.4. Carbon-Based Material Coated Fibers

Among carbon-based materials, graphene-oxide (GO) is widely used to coat optical fibers with the purpose of developing immunosensors with enhanced sensitivity. The structure of this material consists in a 2D honeycomb lattice with carbon atoms, containing functional groups such as hydroxyl, epoxy and carboxyl on its basal plane and sheet edges [111]. As a result, GO can covalently bind to antibodies due to the abundant presence of functional groups on its surface [97]. Therefore, EDC and NHS are used to activate, by cross-linking chemistry, the -COOH functional groups that appear at the edges of GO flakes [111,112]. After activation, the -NH_2_ groups of the antibodies bind covalently with those -COOH groups and generate an amide bond [112].

Another functionalization method uses Protein A before the antibody immobilization, allowing proper antibody orientation as explained above [47,97].

### 3.5. Semiconductor Material Coated Fibers

Semiconductors have already been used to coat sensors, in some cases to trigger an SPR signal, while in others to improve sensitivity by enhancement of the SPR signal characteristics, depending on whether they are deposited directly on the fiber or on previously deposited metal layers. However, their use is still under research.

MoS_2_ is one of the semiconductors that has been investigated and used in immunosensors. The properties of this 2D material make it suitable for the functionalization of sensors since it facilitates a simple and rapid functionalization process. MoS_2_ not only provides higher binding sites for antibody immobilization but also avoids the use of cross-linkers for the biofunctionalization process. In this case, antibodies bind to MoS_2_ through physisorption via hydrophobic interactions [113,114].

ZnO is an oxide semiconductor that has been used to produce immunosensors. One example of the functionalization strategies of this oxide involves its silanization with APTES and subsequent activation with GA. After that process, Protein A can be deposited over the surface of the sensor, followed by the antibody immobilization and surface passivation with BSA [115,116].

Indium tin oxide (ITO) was used in a study developed recently by Maya et al. [98] for IgG detection. In this study, an immunoassay was applied to evaluate the performance of the immunosensor. Consequently, a biolayer composed of a film of IgGs from goat serum was deposited onto the ITO surface. For this to occur, firstly, a polymeric deposition onto the ITO surface was realized with Eudragit L100 (Evonik), which provides -COOH functional groups. The following step was the activation of those groups with EDC and NHS chemistry and the covalent binding of the -NH_2_ groups of the antigens with those -COOH groups [98].

## 4. Biosensing Applications

Over the years, optical fiber immunosensors have emerged as a promising alternative to traditional methods [117,118,119] for the detection of various analytes, including microorganisms, medical biomarkers and environmental pollutants. Moreover, in the specific case of optical fibers as biosensors (e.g., signal response due to RI changes), the molecular size of the analyte could have an influence on the biosensor’s performance, namely in the sensitivity and LOD. Therefore, for low molecular weight analytes, strategies to enhance the signal response are usually applied, for instance using highly sensitive coatings (e.g., gold coating to provide SPR), labels (e.g., fluorescent dyes) or by using competitive approaches in the biosensing setup [120]. There is a panoply of conjugations between fiber configurations and fiber surface coatings, the biofunctionalization procedures employed for the antibody immobilization as well as biosensing setup approach aiming at different applications, as further exemplified throughout this section.

### 4.1. Microorganism Detection

#### 4.1.1. Bacteria

One of the biggest concerns worldwide is related to diseases that are caused by pathogenic bacteria in contaminated food and water [121], mainly in developing countries where the water supply is not clean, the sanitation is poor and malnutrition and overcrowding are present. To tackle this problem more efficiently, several studies have been carried out with the aim of producing immunosensors capable of detecting bacteria while avoiding the usage of complex, time-consuming and expensive strategies such as cell cultures or biochemical assays.

**Figure 11:**
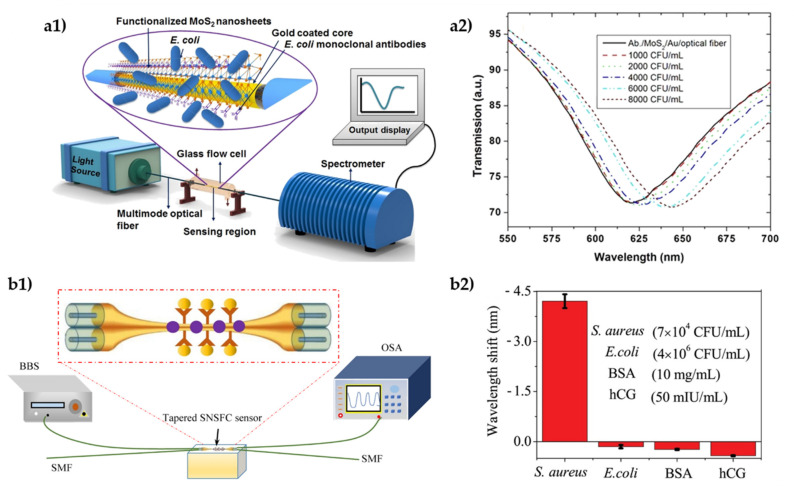
(**a1**) Schematic representation of the setup for the development of MoS_2_ nanosheets-modified, Au-coated, uncladded and etched silica optical fiber for *E. coli* detection, and the anti-*E.coli* antibodies were bonded by hydrophobic interaction to MOS_2_. (**a2**) Transmission spectra as response to *E. coli* concentration. Reprinted with permission from ref. [114]. Copyright 2019 Elsevier. (**b1**) Schematic representation of the setup for the development of tapered single-mode−no-core−single-mode fiber coupler (SNSFC) structure for *S. aureus* detection; the fiber was silanized and the anti-*S. aureus* antibodies were covalently linked using EDC/NHS chemistry. (**b2**) Wavelength shift of the transmission spectra as response to the analyte and the controls. Reprinted with permission from ref. [124]. Copyright 2020 Elsevier, Amsterdam, The Netherlands.

There are various pathogenic bacteria that can appear in contaminated food and water, such as *Escherichia coli* (*E. coli*), namely the serotypes *E. coli O157:H7, E. coli O153:H2* and *E. coli O92: H3*, as well as *Salmonella typhimurium* [114] and *Staphylococcus aureus* (*S. aureus*), to name a few. Several studies have been conducted regarding *E. coli* detection. In 2017, Srinivasan et al. [122] studied the detection of *E. coli* K12 (a non-pathogenic serotype) using a bare FBG sensor functionalized with anti-*E. coli* antibodies. In this case, the detection mechanism was accomplished through an induced strain as a result of the binding of *E.coli* to the specific antibody, thus leading to a Bragg wavelength shift. In the same year, Taniguchi et al. described a fiber optic immunosensor for *E. coli O157:H7* detection. This sandwich approach was performed on an optical fiber with a thin Nickel layer and integrated in a flow cell with a fluorometer coupled to a computer. The sensing signal and reusability of this immunosensor was based on fluorescence and magnetic separation techniques, respectively. The authors detected *E. coli O157:H7* suspension in a range from 1.0 × 10^3^ to 1.0 × 10^7^ cells·mL^−1^, obtaining an LOD of 1.0 × 10^3^ cells·mL^−1^ [122]. In 2018, Kaushik et al. [114] developed a fiber optic SPR immunosensor with MoS_2_ nanosheets, functionalized with anti-*E. coli* antibodies for *E. coli* detection (Figure 11a). In this work, MoS_2_ nanosheets were deposited on an Au-coated etched optical fiber, and then antibodies were immobilized by hydrophobic interactions. Besides, a conventional fiber optic SPR immunosensor was also fabricated to compare with the previously mentioned one. In the former, a range of detection from 1000–8000 colony forming unit (CFU) ·mL^−1^ was achieved with high precision, with an LOD of 94 (CFU)·mL^−1^, the same as above, and a sensitivity of 2.9 nm/1000 CFU·mL^−1^ (3135 nm·RIU^−1^). The conventional fiber optic SPR immunosensor had an LOD of 391 CFU·mL^−1^ and a sensitivity of 0.6 nm/1000 CFU·mL^−1^ (1646 nm·RIU^−1^) [114]. Another study for the detection of *E. coli* was conducted in 2019, in which Kaushik et al. [123] developed an optical fiber immunosensor that consisted of two identical chirped LPGs with an inter-grating space (IGS) of 1 cm, which was functionalized with anti-*E. coli* antibodies. As a result, the grating region of the sensor was not disturbed. This immunosensor worked as a Mach−Zehnder interferometer and the detection mechanism was accomplished through the unique wavelength shifts by changing the RI at the IGS region. In this work, the detection range of this sensor was from 10 to 60 CFU·mL^−1^ and presented an LOD of 7 CFU·mL^−1^ [123].

Regarding *Salmonella typhimurium* detection, in 2018, Kaushik et al. [118] reported a single mode-tapered multimode-single mode (SMS) type of immunosensor. The interaction between the propagating modes of guided light and ambient medium increases due to the tapering of the single-mode. When the interaction increases, the sensitivity of the SMS increases as well. In order to produce the sensing region, some part of the MMF cladding was removed and, afterwards, modified with anti-*Salmonella typhimurium* antibodies. It was possible to detect *Salmonella typhimurium* in a linear range of 500−5000 CFU·mL^−1^, and an LOD of 247 CFU·mL^−1^ was attained along with a sensitivity of 275.86 nm·RIU^−1^ [118].

In 2020, Chen et al. [124] employed a tapered single-mode−no-core−single-mode fiber coupler (SNSFC) structure to produce a label-free optical fiber immunosensor for detection of *S. aureus* bacteria (Figure 11b). This structure allows a much larger taper waist diameter without sacrificing the sensitivity compared with other tapered fiber interferometers. In this case, the no-core fiber was tapered and modified with pig IgG antibodies for *S. aureus* detection. This sensor was tested for different *S. aureus* concentrations ranging from 7 × 10^1^ to 7 × 10^4^ CFU·mL^−1^. The obtained LOD was 3.1 CFU·mL^−1^ [124].

#### 4.1.2. Virus

**Figure 12:**
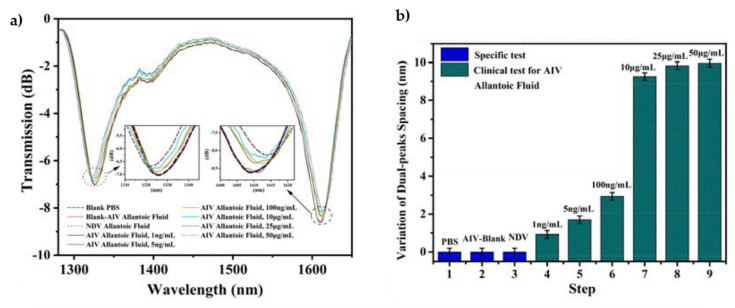
GO-modified turning point LPG for immunodetection of H5N1 virus: (**a**) spectral evolution, the dual-peak movements are magnified in the inset, and (**b**) variations of the dual-peak spacing during the specific tests: PBS, avian influenza virus (AIV) blank and NDV, as well as the clinical tests which consists in AIV allantoic fluid with different concentrations of H5N1 virus. Reprinted with permission from ref. [119]. Copyright 2020 John Wiley & Sons, Inc., Hoboken, NJ, USA.

Early and accurate diagnosis of virus infections is essential to control epidemics and pandemics. The Dengue Virus (DENV) is an arthropod-borne flavivirus that infects millions of people annually. For this reason, Kamil et al. [14] reported a label-free tapered optical fiber immunosensor for detection of DENV E protein, functionalized using APTES as an intermediary linker (Figure 12). The authors achieved a fast detection response of 15 min with a sensitivity of 5.02 nm·nM^−1^ and an LOD of 1 pM. In 2018, Luo et al. [13] developed an immunosensor for the detection of Newcastle disease virus (NDV) based on excessively-tilted fiber Bragg grating (ex-TFBG) coated with Au nanospheres (NSs). The fiber’s surface modified with 3-mercaptopropyltrimethosysilane (MPTMS) was coated with Au NSs via Au-S bonds, while activated Protein A was linked to these NSs through covalent bonds employing cysteamine. The Au NSs enhanced 5–10 times the effect of surrounding RI changes on the fiber cladding mode due to the LSPR effect, by comparison with the fiber without Au NSs. By monitoring the resonance wavelength shifts, the SPR-immunosensor presented an LOD around 25 pg·mL^−1^ and a sensitivity of, approximately, 1.627 pm·(pg·mL^−1^)^−1^ in a linear concentration range of 0–200 pg·mL^−1^ [13]. In 2020, Luo et al. [119] presented an immunosensor based on dispersion turning point LPG incorporating GO to detect H5N1 virus, a common type of avian influenza virus. GO was deposited on the fiber’s surface through hydrogen bonds, whereas anti-H5N1 antibodies were covalently bonded to the GO film by an amide linkage. The immunosensor evidenced a response time from 10 to 20 min and a total variation of the dual-peak spacing of 10.56 nm. The LOD for H5N1 virus was determined to be around 1.05 ng·mL^−1^, which was lower than that of the standard diagnostic method (1.7 µg·mL^−1^) in a detection range from 1 ng·mL^−1^ to 25 µg·mL^−1^ (see Figure 12 [119]). Considering norovirus is the main cause of acute gastroenteritis worldwide, Janczuk-Richter et al. [104] reported an LPG immunosensor capable of detecting norovirus virus-like particles (VLPs) within 40 min. The label-free sensor was tested with norovirus VLP concentrations from 1 ng.mL^−1^ to 10 µg·mL^−1^ and was able to detect concentrations as low as 1 ng·mL^−1^ [104]. In 2021, George et al. [99] presented an immunosensor based on a U-shaped POF using Au NPs as labels to diagnose chikungunya, an infectious mosquito-borne disease. The plasmonic sandwich immunoassay was based on EW absorbance in order to detect the viral protein, chikungunya virus non-structural protein 3 (CHIKV-nsP3). The attained results showed an LOD of 0.52 ng·mL^−1^ (8.6 pM) and a linear range of 1–10^4^ ng·mL^−1^ with a sensitivity of 0.1043 A_530 nm_.(log(C_nsP3_))^−1^, where A_530 nm_ denotes the sensor absorbance response and C_nsP3_ the CHIKV-nsP3 concentration in ng·mL^−1^ [99].

### 4.2. Medical Biomarkers Detection

#### 4.2.1. Cancer Biomarkers

For every disease, early screening and diagnosis significantly improves patient care and leads to higher survival rates. This can be achieved by quantifying disease-specific biomarkers resorting to portable and user-friendly optical fiber immunosensors, which enable POC testing near the patient and in real time. Over the years, there has been an increase of reported diagnostic devices for biomarker detection, with cancer being one of the most common targets.

**Figure 13:**
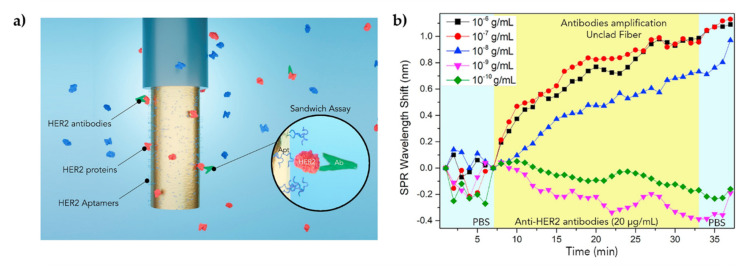
Sandwich assay for detection of the HER2 as cancer biomarker: (**a**) gold-coated unclad fiber functionalized with thiolated aptamers specific to recognize HER2 and further signal amplification using anti-HER2 antibodies; (**b**) SPR wavelength shift as response to HER2 concentration using anti-HER2 antibodies for signal amplification. Reprinted with permission from ref. [44]. Copyright 2021 Elsevier.

Loyez et al. [44] described a sandwich optical fiber assay to detect HER2 breast cancer biomarker based on SPR using an unclad end-face reflected biosensor. In this research, the cladding was removed from the fiber tip (1 cm length), which was then coated with an Au film via sputtering to enhance the sensitivity towards RI changes. The experimental setup consisted of a white light source and a spectrometer both connected to the sensor that guided light to the fiber tip, where it was reflected back towards the spectrometer. The performance of direct (label-free) and indirect approaches for detecting HER2 proteins was evaluated. In both cases, thiolated anti-HER2 aptamers were directly linked to the Au film surface. However, the indirect approach involved a signal amplification by further immobilization of anti-HER2 antibodies in a sandwich layout (aptamers-HER2-antibodies); see Figure 13. By monitoring wavelength shifts of the SPR minimum in the spectral response, the biosensor demonstrated a sensitivity of 0.17 nm·nM^−1^. Moreover, the sandwich assay proved to lower the LOD of HER2 biomarkers from 0.6 µg·mL^−1^ to 9.3 ng·mL^−1^ compared to label-free method [44]. Sun et al. [93] also proposed another HER2 biomarker immunosensor, using a fiber optic taper interferometer cascaded with an FBG. The FBG, which is insensitive to RI changes, was used only as a temperature thermometer, whereas HER2 biomarker detection was carried out by the functionalized tapered fiber optic interferometer. The obtained results evidenced an LOD of 2 ng·mL^−1^, having tested HER2 concentrations between 2 and 100 ng·mL^−1^ [93]. In 2017, Ribaut et al. [125] reported an innovative Au coated-TFBG immunosensor for the detection of CK17, a biomarker of interest for lung cancer diagnosis. Here, TFBGs with a 7° tilt angle and a 50 nm Au coating were immersed in S_2_-PEG_6_-COOH to create a self-assembly monolayer, followed by immobilization of AbCK17 on the surface of the TFBGs. With the purpose of developing a sensing platform that can assess non-liquid environments, detections were made on CK17 encapsulated in gel matrix that were mimicking tissue samples. The sensor could selectively detect CK17 with an LOD of 0.1 ng·mL^−1^. Then, the fiber sensor was packed to be utilized for ex vivo testing of a human lung biopsy and successfully distinguished samples of healthy tissue from tissue with tumors. The LOD of CK17 antigen could reach 0.4 nM based on the same sensing platform. According to the authors, this work represented an important milestone towards the detection of biomarkers in tissues [125]. A sensitive PCF-based immunosensor was proposed by Liu et al. [107] for the detection of alpha fetoprotein (AFP), an oncofetal glycoprotein and a biomarker for hepatocellular carcinoma. The surface of the silica PCF was pre-treated with ADMS and GA, followed by covalent immobilization of the antibody, which was used to capture the AFP human antigen that in turn captured the Alexa Fluor 488-labeled AFP secondary antibody. The sensor response was measured by the laser-induced fluorescence detection system. The results demonstrated a linear dynamic range from 0.1 to 150 ng·mL^−1^ and an LOD of 0.1 ng·mL^−1^, a value that was lower than that of ELISA (3.5 ng·mL^−1^) and below the accepted decision threshold for AFP in serum to establish disease diagnosis (10 ng·mL^−1^) [107]. In 2019, Luo et al. [126] resorted to ex-TFBG coated with Au nanoshells for label-free detection of soluble programmed death ligand-1 (sPD-L1). sPD-L1 portrays an essential role in tumor evasion from the immune system and therefore the level of sPD-L1 in serum is indicative of tumor aggressiveness and outcome. In this research, anti-sPD-L1 antibodies were attached to the LSPR-based ex-TFBG surface employing Protein A. The immunosensor evidenced an LOD of ∼1 pg·mL^−1^ in buffer solutions and of 5 pg·mL^−1^ in complex serum media, such as fetal bovine serum (FBS) [126].

#### 4.2.2. Cardiac Biomarkers

**Figure 14:**
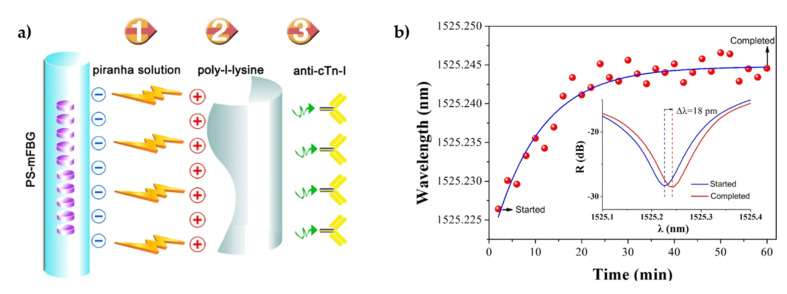
Phase-shifted microfiber Bragg grating (PS-mFBG) functionalized for specific detection of cTn-I as a cardiac biomarker: (**a**) PS-mFBG functionalized via electrostatic interactions, (1) using piranha solution yielding a negative surface and then (2) using poly-l-lisine as a positive polymer intermediary layer where the (3) anti-CTn-I antibodies with the opposite surface charge physically adsorbed via electrostatic interactions; (**b**) wavelength variation of the notch, represented in the inset, as function of time during the specific binding recognition. Reprinted with permission from ref. [109]. Copyright 2018 Elsevier.

Cardiovascular diseases, including acute myocardial infarction (AMI) and heart failure (HF), are a major cause of morbidity and mortality across the world [127]. Hence, several cardiac biomarkers have been researched through the years for early screening of these diseases, including cardiac troponin I (cTnI), an indicator of AMI, and Human N-terminal pro-B-type natriuretic peptide (NT-proBNP), an HF biomarker. In 2018, Liu et al. [109] proposed a cTnI label-free immunosensor deploying a phase shifted microfiber Bragg grating (PS-mFBG), as represented in Figure 14. The π-phase-shift in modulation granted a narrow band notch signal in the reflection spectrum of the mFBG, drastically enhancing sensor resolution. The sensing mechanism was based on monitoring the wavelength shift of the notch in the reflective spectrum, caused by changes of the RI surface because of the antigen-antibody binding. The use of a tapered microfiber (3.5 μm fiber diameter) enhanced the evanescent field on the fiber surface and thus enabled a greater interaction between the transmitted light and the surrounding medium. This biosensor obtained a log-linear sensing range from 0.1 to 10 ng·mL^−1^ and an LOD of 0.03 ng·mL^−1^ [109]. Another label-free immunosensor for cTnI detection was designed by Zhou et al. [127] based on an optical microfiber coupler (OMC). Here, the immobilization of cTnI antibodies was realized via polyelectrolyte layer using a layer-by-layer deposition method. The best results were achieved when the OMC was operating near the dispersion turning point, obtaining an LOD of 2 fg·mL^−1^ in PBS buffer and a linear wavelength shift in the 2–10 fg·mL^−1^ range [127]. In 2017, Luo et al. [128] developed a highly specific immunosensor platform using ex-TFBGs (tilt angle > 80°) for fast and label-free detection of NT-proBNP. The immunoassays were performed in vitro in human serum samples by monitoring the shift of the resonance wavelength, with a fiber optic grating demodulation system, once in the presence of NT-proBNP antigens. The attained results indicated an LOD around 0.5 ng·mL^−1^ and an average sensitivity of 45.967 pm·(ng·mL^−1^)^−1^ at a concentration range of 0.0–1.0 ng·mL^−1^. This low linear dynamic range was attributed to the small active length of the sensor; therefore, the authors postulated that a longer TFBG immunosensor could, theoretically, lead to an increase in the linear dynamic range [128].

#### 4.2.3. Stress Biomarkers

When stress is persistent and uncontrollable, it is considered pathological, which can trigger depression and cardiovascular diseases [129], for example. Therefore, the development of technology capable of monitoring stress is essential. Stress involves a large number of neuronal circuits and, once it is promoted, leads to the release of glucocorticoids, in particular cortisol [130]. The substantial variation of this hormone occurs due to exposure to psychological, environmental or emotional stress [131]. As a result, cortisol is one of the most important stress biomarkers.

One area where stress has also presented a lot of influence is aquaculture, whose production has been widely performed in recirculating aquaculture systems. In these systems, when small variations in water chemistry or quality occur, stress induction can arise, as well as reduced food intake that leads to reduced fish growth and can also lead to mortality when acute or chronic stress is high. In this sense, it is also essential to monitor cortisol in water [132,133].

**Figure 15:**
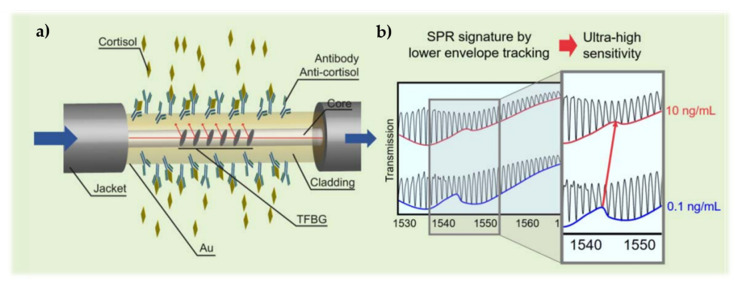
SPR-based TFBG developed for specific detection of cortisol as a stress biomarker: (**a**) Au thin film on the TFBG was modified with cysteamine and then anti-cortisol antibodies were covalently bonded via EDC/NHS chemistry; (**b**) representation of the SPR signature spectra variation and the corresponding lower envelope as a response to two different cortisol concentrations. Reprinted with permission from ref. [92]. Copyright 2021 IEEE, Piscataway, NJ, USA.

In 2020, Sharma et al. [134] simulated an SPR fiber optic immunosensor for salivary cortisol detection at the wavelength of 830 nm. The sensor presented an Ag layer with 2D materials, conventional (graphene, tungsten disulfide (WS_2_) and MoS_2_) and transition metal carbides (MXenes: Ti_3_C_2_, Ti_3_C_2_O_2_, Ti_3_C_2_F_2_, Ti_3_C_2_(OH)_2_) considered one at a time, which operated in two modes (‘AIM’ and ‘IIM’). The sensor that showed a superior balanced set of performance parameters under both modes was the Ti_3_C_2_O_2_-based probe. Through the simulation, this probe was able to achieve an LOD of 15.7 fg·mL^−1^ [134]. Recently, Leitão et al. [84] reported an SPR unclad POF immunosensor coated with gold/palladium (AuPd) alloy. This sensor was modified with anti-cortisol antibody for cortisol detection. The detection mechanism relied on the shift of the SPR wavelength achieved through the RI variation on the AuPd surface, due to the antibody−antigen binding reaction. For a range from 0.005 to 10 ng·mL^−1^ of cortisol concentration, the proposed sensor had a 15 nm wavelength shift, allowing to conclude that this sensor presented high sensitivity. The attained sensitivity and LOD were 3.56 ± 0.20 nm·(log(ng·mL ^−1^))^−1^ and 1 pg·mL^−1^, respectively. In this research, selectivity tests were also performed in a sensor functionalized with antibodies for human chorionic gonadotropin (anti-hCG antibodies) in which the variance of the resonance wavelength was only 1 nm, much lower than the sensor modified with anti-cortisol antibodies [84]. In another recent study, Leitão et al. [92] developed and tested an Au-coated TFBG immunosensor based on SPR, functionalized with anti-cortisol antibodies for cortisol detection (see Figure 15). In this investigation, an alternative interrogation method was employed. Hence, the local maximum of the plasmonic signature of the lower envelope of the spectra was signalized to monitor the SPR mode. This sensor was tested for a linear cortisol concentration range of 0.1–10 ng·mL^−1^, obtaining a total wavelength shift of 3 nm and a sensitivity of 0.275 ± 0.028 nm·(ng·mL^−1^)^−1^ [92].

#### 4.2.4. Inflammatory Biomarkers

The detection of inflammatory biomarkers is crucial for obtaining early disease diagnosis, screening diseases and even for monitoring treatment efficacy [135]. The development of diagnostic devices for inflammatory biomarkers detection has been growing recently.

For instance, Liu et al. [136] demonstrated a cytokine interleukin-6 (IL-6) detection device in 2016. This device consisted of a sandwich immunoassay scheme with an SOF coated with Au NPs and, in turn, functionalized with IL-6 antibodies. The fiber was introduced into an intrathecal catheter with microscopic holes to allow the exchange of a fluid between the outside and the inside of the catheter. After the contact with IL-6, the sensor was exposed to the IL-6 antibody marked with fluorescently-labeled magnetic NPs in order to quantify the cytokine concentration through fluorescence intensity. The attained LOD of this sensor was 1 pg·mL^−1^ for a linear detection range of 1–400 pg·mL^−1^ [136].

**Figure 16:**
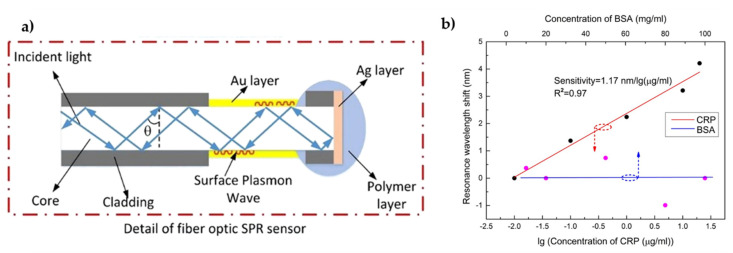
SPR optical fiber for specific detection of CRP: (**a**) representation of the sensing system in a reflected mode: the Au layer was modified with polydopamine as intermediary layer where the anti-CRP antibodies were bonded; (**b**) wavelength shift as response to CRP concentration; a control experiment using BSA is also displayed. Adapted from ref. [30].

In 2017, a plasmonic optical fiber immunosensor was developed by Wang et al. [30] for specific detection of C-reactive protein (CRP). In this sensor, an Au layer was deposited on the unclad sensing region at one end of the fiber, as well as an Ag reflector on the end face of the fiber and a polymer-covering layer to prevent Ag oxidation (Figure 16a). The biological modification of the sensor surface involved a polydopamine film acting as a cross-linking agent, followed by the immobilization of anti-CRP antibodies. The immobilization time of antibodies and the reaction time between antigen and antibody were both studied and optimized experimentally, leading to a linear response within the 0.01–20 µg·mL^−1^ range and a CRP sensitivity of 1.17 nm per log (µg·mL^−1^). This sensitivity was determined as the resonance wavelength shift per unit change in the logarithm of CRP concentration. In addition, specificity was analyzed by comparing the resonance wavelength shift difference in BSA and in CRP detection, with the former being significantly smaller (Figure 16b) [30].

More recently, in 2020, Nie et al. [135] reported a portable pencil-like immunosensor platform, or in other words, a portable and versatile chemiluminescence-based optical fiber immunosensor for the detection of IL-6, procalcitonin (PCT) and CRP in human serum samples. This platform consisted of a unique pencil-like optical fiber-based sensor, a reagent strip consisting of a series of pencil-cap-like wells and a battery-powered photon counting detector for recording chemiluminescence. Hence, this platform combined an immunosensor with immunoassay process, chemiluminescence detection and data analysis in a portable suitcase-like device. A silica fiber was used, and to immobilize the biorecognition molecules, some part of the cladding on the distal end was removed. This sensor presented an LOD for IL-6, PCT and CRP of 1.05 pg·mL^−1^, 10.64 pg·mL^−1^ and 29.40 ng·mL^−1^, respectively, and an excellent linear relationship from 5 to 10,000 pg·mL^−1^ for IL-6, from 0.05 to 200 ng·mL^−1^ for PCT, and from 0.1 to 80 μg·mL^−1^ for CRP. After 14 days of storage at room temperature, this sensor still maintains 90 % of response, which is a benefit for field assays [135].

### 4.3. Environmental Monitoring

Over the years, there has been an increasing concern towards emerging contaminants and pollutants as they can severely affect the environment and human health. These contaminants, which include antibiotics, anti-inflammatory drugs, illicit drugs and stimulants, are biologically active and highly resistant to degradation. Therefore, they persist in the environment, causing harmful effects to nontarget organisms and humans [137]. For this reason, methods capable of accurately detecting these substances are imperative to protect both the environment and human health.

**Figure 17:**
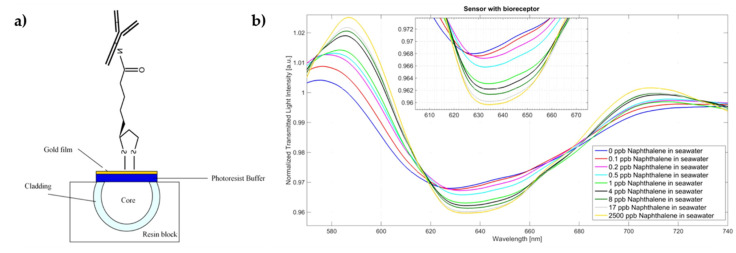
(**a**) Representation of the SPR-based D-shaped POF sensor for specific detection of naphthalene; the Au thin layer was modified with α-lipoic acid and then the anti-naphthalene antibodies were covalently linked via EDC/NHS chemistry; (**b**) normalized transmitted spectra in the wavelength range of the SPR showing its variations as a response to the naphthalene concentration. Reprinted with permission from ref. [56]. Copyright 2019 Elsevier.

Phthalate esters (PAEs) are a type of synthetic chemical substance that have been allocated to the list of priority pollutants, as a result of their endocrine-disrupting and toxic effects on the human body. In 2017, Tang et al. [103] proposed an optical fiber immunosensing platform for rapid detection of PAEs in greenhouse soils based on indirect competitive immunoassay. Coating antigen was covalently immobilized on the sensor surface, with its binding reaction to fluorescent-labeled antibody creating an inhibition signal of PAEs that was detectable by an avalanche diode. The developed immunosensor had a linear range of 0.01–100 µg.L^−1^, and the limits of detection of eight PAEs ranged from 19 ng·L^−1^ to 51 ng·L^−1^. The average recoveries of PAES at the spiked levels of 5, 10 and 100 µg·kg ranged between 61.5% and 106.7%, with relative standard deviations below 13.41% [103]. In 2019, Cennamo et al. [56] developed and characterized a D-shaped plasmonic optical fiber biosensor to detect the presence of naphthalene (NAPHTA) in sea water. For this purpose, an Au layer (10 nm thickness) was deposited by sputtering on the surface of a POF to allow SPR. Then, a designed and produced antibody specific to the NAPHTA molecule was covalently linked through EDC/NHS chemistry to the Au surface modified with α-lipoic acid (Figure 17). Tests using real matrices of sea water were performed using the produced biosensor. The POF biosensor was able to sense the presence of NAPHTA in sea water solution with an LOD of 0.76 ng·mL^−1^, which is lower than the limit value of NAPHTA (0.13 μg·mL^−1^) fixed by European Union regulations [56]. In 2021, a U-shaped fluorescent immunosensor was designed by Liu et al. [138] for real-time detection of microcystin-LR (MC-LR), a widespread cyanotoxin with high toxicity, in water samples. In this work, light-sheet was used for skew ray excitation in order to enhance sensitivity by enhancing the light−matter interactions. From the experimental results, an LOD of 0.46 µg·L^−1^ was calculated, with a linear range between 0.84–6.50 µg·L^−1^ [138]. Yang et al. [139] also focused on the detection of MC-LR in water samples by developing a portable and reusable chemiluminescent immunosensor. Considering MC-LR possesses a low molecular weight and, therefore, is difficult to immobilize directly onto the sensor surface, hapten-carrier protein conjugates were covalently immobilized on the aminated fiber surface. Under optimal conditions, the LOD of MC-LR was 0.03 µg·L^−1^. Moreover, the linear response ranged from 0.23 to 190 µg·L^−1^ [139]. In a 2020 study, Lamarca et al. [137] prepared a label-free U-shaped immunosensor for the determination of ciprofloxacin (CIP) in wastewater samples, seeing as though CIP is a broadly utilized antibiotic to treat infections and is a common contaminant of wastewater treatment plants. The glass optical fiber surface was functionalized with polyaniline, followed by immobilization of anti-CIP IgG. CIP could be detected with an LOD of 3.30 × 10^−3^ ng·L^−1^ in a linear range between 0.01 and 10,000 ng·L^−1^. Moreover, the immunosensor offered a high average recovery of 91 % [137]. Chang et al. [110] reported an optical fiber LSPR immunosensor for rapid and sensitive detection of MA in urine samples. In this research, Au NPs were self-assembled on the unclad portion of an optical fiber, and then the surface of these NPs was functionalized with BSA-MA. The aim of this study was to detect, with high sensitivity, MA at ultra-low concentration using a competitive inhibition immunoreaction scheme based on the competition of free MA in a solution for anti-MA antibodies. As a result, in the presence of a fixed concentration of anti-MA antibodies, once the MA concentration increases, the change of transmitted light intensity through the sensor fiber relative to that in a buffer solution decrease. Based on this sensing technique, the immunosensor presented not only a wide linear dynamic range of 1–1000 ng·mL^−1^ with an LOD of 0.16 ng·mL^−1^, but also could detect MA in human urine samples without any nonspecific adsorption interference [110]. Benzo[a]pyrene (B[a]P) is considered one of the most toxic polycyclic aromatic hydrocarbons and a carcinogen, making monitoring its concentration levels essential for human health and environmental contamination. To monitor B[a]P contamination levels in water, Gao et al. [140] proposed an in-line fiber optofluidic immunosensor using a hollow-core fiber. As such, the immunoreaction between the antibody and the B[a]P molecule induced a significant change of the RI inside the in-line optofluidic channel. The attained results presented an LOD of 1.65 pM and sensitivities of up to 23 pm·pM^−1^ [140].

A plethora of other optical fiber-based immunosensors have been reported in recent years for different applications, including detection of mycotoxins relevant for food safety [115,116,141] and BSA [113,142], among others. In Table 2, a summary of the immunosensors described is presented, highlighting the main figures of merit.

## 5. Final Remarks and Future Outlook

This paper presents a review on optical fiber-based biosensors, covering different geometries and biofunctionalization strategies along with practical application examples. The use of optical fiber technology has increased tremendously over the years owing to unique characteristics of these waveguides such as small size, flexibility and immunity towards electromagnetic interference, among others. While unmodified optical fibers dominate the field of telecommunications, several geometrical alterations have been investigated for biosensing purposes. These modifications can rely on bending, tapering, removing the cladding or inscribing fiber gratings in the core and so forth, leading to different detection mechanisms, based on light transmission or reflection. Furthermore, recent developments regarding special fibers such as MOFs and PCFs have attracted great interest as these provide an effective and long-distance interaction between light and the analytes of interest.

Optical fiber biosensors allow for sensor miniaturization as well as the ability for in situ and real-time monitoring. Hence, they have been deployed in various fields for different applications and can be distinguished according to the biofunctionalization method and the type of biorecognition molecule. Direct biofunctionalization methods employ uncoated modified fibers where the EW interacts with sample analytes. On the other hand, plasmonic biosensors require a coating material for either SPR or LSPR to occur, with the latter relying on the addition of metal NPs, which provide a large surface contact area. These plasmonic biosensors benefit from being a label-free method to detect biomolecular interactions with high sensitivity and low LOD. While Au and Ag are predominantly applied as surface coatings, seeing as though their high sensitivity has been broadly demonstrated, the addition of graphene and GO layers has proven to increase sensitivity. Still, the inclusion of other materials, ranging from semiconductors to oxides and nitrides, may be the future direction of this field. Regarding the biorecognition molecule, immunosensors stand out due to their high sensitivity, specificity and versatility as a result of the great bioaffinity between antibodies and the corresponding antigens. 

Enhancing sensor performance is a constant process that will continue to drive researchers to improve, namely, repeatability and reproducibility, as it requires great control over the fabrication and functionalization procedures of the sensor. While optical fiber biosensors are still limited in clinical setting, their promising results and continued investment towards improvement will likely prompt them to transition from laboratory environments to commercial applications in the future. In this sense, due to the miniaturization of optical fibers, their use for in vivo applications in hard-to-reach locations is a possibility [143]. In addition, another future direction may rely on incorporating artificial intelligence software to facilitate signal acquisition and data analysis [42]. Moreover, sensor regeneration and reusability [13,75,139] pose a promising prospect for future improvement and lower costs in this field. The demand for reusable biosensors arises from benefits over their disposable counterparts, namely lower costs associated with materials and reagents, in addition to reduced preparation time since the attained calibration curve is suitable for several subsequent measurements. This ensures improved detection speed and reproducibility, while also enabling a large number of samples to be tested semi-continuously [144]. However, regenerating an immunosensor relies on detaching antigens from the antibodies on the fiber surface, a process that can damage the antibodies and hinder repeated usage. Therefore, while there is already some research regarding sensor regeneration and reusability using acid solutions [13,127,144,145], these remain a challenge for the future. In addition, the ability for detection of various parameters within a single optical fiber device [85,146] is another challenge to overcome in the promising future of biosensing.

Enormous scientific and technological advances were made in optical fiber immunosensors in recent years. Nonetheless, similarly to other biosensing techniques, they are still underused at the commercial level. This can be explained by some challenges that are still unresolved in the area of many types of biosensors, particularly immunosensors. Currently, it is still difficult to produce large-scale, robust, reliable and highly repeatable devices, as well as to develop data acquisition modules that meet POC requirements. Within the next few years, advances in material laser processing and low-cost interrogation techniques will certainly promote the translation of these devices to the market. Finally, stability and lifetime are two important criteria to bear in mind regarding commercialization. In a laboratory scale, stability and storage of biosensors are easier to control and assess. Besides, their preparation and application are usually in a range of days, sometimes weeks, under controlled temperature and humidity environments. However, stability as well as storage and operation lifetime have to be carefully evaluated regarding real-life applications. The time between production and real use can be in the order of months, and thus, careful attention should be placed on the storage, transportation and operation conditions in order to keep the performance of the biosensors reliable, which in many cases remains a challenge.

## Figures and Tables

**Table 1:** Figures-of-merit of biosensors.

Figures-of-Merit	Definition
Linear concentration range	Detection range within the sensor has a linear response
Sensitivity (*S*)	Ratio between the change in output signal in response to variations of the concentration of the measured analyte
LOB	Highest apparent analyte concentration expected to be found when replicates of blank samples are tested (no analyte in the samples)
LOD	Lowest analyte concentration, which is not related to noise, detectable by the sensor with acceptable degree of certainty
LOQ	Lowest analyte concentration that can be quantified with an acceptable degree of certainty
Specificity	Ability to discern the response of the analyte of interest from every other interferent
Accuracy	Proximity between the sensor output value and the actual value of the measured analyte
Reproducibility	Capability to obtain a similar response over several repetitions using the same sensor and analyte when performing under different conditions
Repeatability	Capability to obtain a similar response over several repetitions using the same sensor and analyte when performing under the same conditions

**Table 2:** Performance parameters and characteristics of state-of-the-art immunosensors.

Sensor Structure	Analyte	Surface Coating	Detection Principle	Sensitivity	LOD	Linear Range	Ref.
**Bacteria**							
FBG	*E. coli*	-	Strain	-	-	-	[101]
POF	*E. coli O157:H7*	-	Fluorescence	-	1.0 × 10^3^ cells·mL^−1^	1.0 × 10^3^–1.0 × 10^7^ cells·mL^−1^	[122]
Etched POF	*E. coli*	Au and MoS_2_	SPR	2.9 nm/1000 CFU·mL^−1^ (3135 nm·RIU^−1^)	94 CFU·mL^−1^	1000–8000 CFU·mL^−1^	[114]
CLPG	*E. coli*	-	RI	-	7 CFU·mL^−1^	10–60 CFU·mL^−1^	[123]
SMS	*Salmonella Typhimurium*	-	RI	275.86 nm·RIU^−1^	247 CFU·mL^−1^	500–5000 CFU·mL^−1^	[118]
Tapered SNSFC	*S. aureus*	-	RI	-	3.1 CFU·mL^−1^	7 × 10^1^–7 × 10^4^ CFU·mL^−1^	[124]
**Virus**							
Tapered SMF	DENV E proteins	-	RI	5.02 nm·nM^−1^	1 pM	-	[14]
LPG	H5N1	GO	RI	-	1.05 ng·mL^−1^	1 ng·mL^−1^–25 μg·mL^−1^	[119]
U-shape POF	CHIKV-nsP3	-	RI	0.1043 A_530_ nm·(log(C_nsP3_))^−1^	0.52 ng·mL^−1^	1–10^4^ ng·mL^−1^	[99]
Ex-TFBG	NDV	Au nanospheres	LSPR	-	25 pg·mL^−1^	0–200 pg·mL^−1^	[13]
LPG	Norovirus VLP	-	RI	-	1 ng·mL^−1^	-	[104]
**Cancer Biomarkers**							
Unclad end-face reflected	HER2	Au	SPR	0.17 nm·nM^−1^	9.3 ng·mL^−1^	-	[44]
Taper interferometer cascaded with FBG	HER2	-	RI	-	2 ng·mL^−1^	-	[93]
TFBG	CK17	Au	SPR	-	0.1 ng·mL^−1^ (gel matrix)	-	[125]
PCF	AFP	-	Fluorescence	-	0.1 ng·mL^−1^	0.1–150 ng·mL^−1^	[107]
ex-TFBG	sPD-L1	Au nanoshells	LSPR	-	1 pg·mL^−1^ (buffer) 5 pg·mL^−1^ (FBS)	-	[126]
**Cardiac Biomarkers**							
PS-mFBG	cTnI	-	RI	-	0.03 ng·mL^−1^	0.1–10 ng·mL^−1^	[109]
OMC		-	RI	-	2 fg·mL^−1^	2–10 fg·mL^−1^	[127]
Ex-TFBG	NT-proBNP	-	RI	45.967 pm.(ng·mL^−1^)^−1^	0.5 ng·mL^−1^	0.0–1.0 ng·mL^−1^	[128]
**Stress Biomarkers**							
Unclad POF	Cortisol	Ag and Ti_3_C_2_O_2_	SPR	-	15.7 fg·mL^−1^	0.36–4.50 ng·mL^−1^	[134]
Unclad POF		Au/Pd	SPR	3.56 ± 0.20 nm·(log(ng·mL^−1^))^−1^	1 pg·mL^−1^	0.005–10 ng·mL^−1^	[84]
TFBG		Au	SPR	0.275 ± 0.028 nm·(ng·mL^−1^)^−1^	-	0.1–10 ng·mL^−1^	[92]
**Inflammatory Biomarkers**							
SOF	IL-6	Au NPs	Fluorescence	-	1 pg·mL^−1^	1–400 pg·mL^−1^	[136]
Unclad End-face reflected	CRP	Au	SPR	1.17 nm·(log(µg·mL^−1^))^−1^	-	0.01–20 µg·mL^−1^	[30]
PPS (End-face reflected)	IL-6	-	Chemiluminescence	-	1.05 pg·mL^−1^	5–10,000 pg·mL^−1^	[135]
	PCT				10.64 pg·mL^−1^	0.05–200 ng·mL^−1^	
	CRP				29.40 ng·mL^−1^	0.1–80 μg·mL^−1^	
**Environmental**							
End-face reflected	PAEs	-	Fluorescence	-	19–51 ng·L^−1^	0.01–100 µg·L^−1^	[103]
D-shape	NAPHTA	Au	SPR	-	0.76 ng·mL^−1^	-	[56]
U-shape	MC-LR	-	RI Fluorescence	-	0.46 µg·L^−1^	0.84–6.50 µg·L^−1^	[138]
SOF		-	Chemiluminescence	-	0.03 µg·L^−1^	0.23–190 µg·L^−1^	[139]
U-shape	CIP	-	RI	-	3.30 × 10^−^^3^ ng·L^−1^	0.01–10,000 ng·L^−1^	[137]
Unclad	MA	Au NPs	LSPR	-	0.16 ng·mL^−1^	1–1000 ng·mL^−1^	[110]
Hollow-core fiber	B[a]P	-	RI	23 pm·pM^−1^	1.65 pM	-	[140]

## Data Availability

The data presented in this study are available on request from the corresponding author.
